# Preventive health service coverage among infants and children at six maternal-child health clinics in western Kenya: A cross-sectional assessment

**DOI:** 10.1007/s10995-021-03271-8

**Published:** 2021-10-29

**Authors:** Andrew Deathe, Eren Oyungu, Samuel O. Ayaya, Ananda R. Ombitsa, Carole I. McAteer, Rachel C. Vreeman, Megan S. McHenry

**Affiliations:** Centers for Disease Control and Prevention; Moi University; Moi University; Moi University; Indiana University Department of Pediatrics; AMPATH Kenya; Indiana University Department of Pediatrics

**Keywords:** child health, health service coverage, Kenya, sub-Saharan Africa, immunizations, maternal and child health

## Abstract

**Background:**

Despite the substantial reduction of child mortality in recent decades, Kenya still strives to provide universal healthcare access and to meet other international benchmarks for child health. This study aimed to describe child health service coverage among children visiting six Maternal and Child Health (MCH) clinics in western Kenya.

**Methods:**

In a cross-sectional study of Kenyan young children (≤5 years) presenting to MCH clinics, child health records were reviewed to determine coverage of immunizations, growth monitoring, vitamin A supplementation, and deworming. Among 78 children and their caregivers, nearly 70% of children were fully vaccinated for their age.

**Results:**

We found a significant disparity in full vaccination coverage by gender (p = .017), as males had 3.5x higher odds of being fully vaccinated compared to females. Further, full vaccination coverage also varied across MCH clinic sites ranging from 43.8% to 92.9%.

**Conclusions:**

Health service coverage for Kenyan children in this study is consistent with national and sub-national findings; however, our study found a significant gender equity gap in coverage at these six clinics that warrants further investigation to ensure that all children receive critical preventative services.

## Background

In 2016, nearly six million children under five years of age globally died (1). Despite being home to nearly 14% of the world’s population, sub-Saharan Africa (SSA) accounts for nearly 3.2 million of these deaths (1). Further, the under-five mortality rate in Kenya is 49 deaths per 1,000 live births (1). The most significant causes of mortality in children under five years of age in Kenya are preventable or treatable conditions (2). In Kenya, Maternal-Child Health (MCH) clinics are the primary source of healthcare for infants. MCH clinics provide services to prevent and treat diseases, including immunizations, vitamin A supplementation, deworming, and growth monitoring, as well as serve as a source of educational knowledge about health-related topics. Inadequate provision of or access to these services during the prenatal, perinatal, and early childhood periods contributes to unnecessary infant and child deaths (3). A previous study found that increasing interventions and services within the maternal and child health care continuum are largely responsible for the decline in childhood mortality. Therefore, monitoring coverage of these interventions and services is critical to determine gaps and barriers affecting service provision (4).

In Kenya, preventative services, such as immunizations, vitamin A, and deworming, are reported on national and regional levels within the Kenya Demographic and Health Survey (KDHS), most recently published in 2015 (5). However, national coverage estimates may not accurately depict situations at the local levels, due to population-based sampling. To achieve the ambitious targets of the Sustainable Development Goals, high quality preventive healthcare services must be accessible for all children. Therefore, it is necessary to address inequalities and disparities affecting the access, utilization, delivery, and subsequent coverage of services provided to children. Identifying the services that are provided at these MCH clinics, the coverage of services, and any gaps that may prevent children from receiving the critically important care is a crucial first step in moving Kenya forward in the new Sustainable Development Goals era.

Within the health records, Kenya’s Ministry of Health prioritizes weight and height measurements, immunizations, deworming, and vitamin A supplementation as key areas for preventative healthcare for children under the age of five years. Therefore, we assessed these key areas in this study (6). The objective of the study was to determine the coverage of services and interventions provided to infants and children at six maternal and child health clinics in western Kenya in order to identify gaps in the services with the ultimate goal of identifying factors that may be associated with lower coverage.

## Methods

This cross-sectional descriptive study was performed at six MCH clinics located within Bungoma, Nandi, Trans Nzoia, and Uasin Gishu counties located in western Kenya. The clinics are located in the towns of Eldoret, Turbo, Webuye, Mosoriot, Burnt Forest, and Kitale. These Ministry of Health clinics work in close collaboration with an institutional partnership: Academic Model Providing Access to Healthcare (AMPATH). This institutional partnership isled by Moi University School of Medicine and Indiana University and aims to improve delivery of healthcare services in western Kenya (7). This existing partnership and their close collaboration with the local MCH clinics throughout the region provided the rationale for the selection of the study setting. This study was approved by the ethical committees of both Indiana University and Moi University. Moi University’s Institutional Research and Ethics Committee is registered with the U.S. Office of Human Research Protections with its own Federalwide Assurances.

From 11/7/2016 – 12/7/2016, caregivers were recruited for participation at six MCH clinic locations using convenience sampling. A research assistant attended a full pediatric clinic day and approached all caregivers meeting the following inclusion criteria: bringing a child under the age of five years to be seen and identifying their current location as their primary MCH clinic. Every present caregiver who met inclusion criteria was recruited. A total of 78 caregivers consented and agreed to participate in the study. Only one recruited caregiver declined participation. For each caregiver, the child brought to clinic was also included in the study. Only retrospective data were collected from the children’s medical record.

The Mother and Child Health Booklet was the primary data source for this study. This booklet contains the services and interventions received by the child at the MCH clinic. Kenya began using The Mother and Child Health Booklet in 2008 to link maternal and child healthcare and have one comprehensive medical record (6). The Mother and Child Health Booklet provides education on strategies to improve her and her child’s health. The book, which is the primary health record for the mother and her child, is brought to every visit with a healthcare provider. Healthcare providers record services and interventions provided at each visit in the booklet.

In Kenya, children are immunized against tuberculosis, diphtheria, whooping cough (pertussis), tetanus, polio, measles, hepatitis B, *Haemophilus influenza* type b (Hib), *Streptococcus pneumonia*, and rotavirus (5). Bacillus Calmette-Guerin (BCG) is used for immunization again tuberculosis. The pentavalent vaccine provides protection against diphtheria, pertussis, tetanus, hepatitis B, and Hib. The administration of the oral polio vaccine (OPV) immunizes again poliovirus, and measles and rotavirus each have their own vaccine. Lastly, children are vaccinated against Streptococcus pneumoniae via administration of the pneumococcal conjugate vaccine (PCV) (5). Among the six vaccines included in the routine immunization schedule in Kenya, all but one, BCG, is given in a series of multiple doses.

To prevent soil-transmitted helminth infections, which are associated with malnutrition, poor physical growth, and cognitive impairment (8, 9), the World Health Organization and Kenya’s Ministry of Health recommends children ages 12–59 months receive one dose of either albendazole or mebendazole every 6 months. Additionally, vitamin A deficiency affects nearly 30% of children in low- and middle-income countries and is linked to child mortality (10). To prevent vitamin A deficiency, high-dose vitamin A is given once every 6 months as a supplement, beginning at 6 months of age and ending at 59 months of age (5).

For data collection, brief oral interviews with caregivers and review of the Mother and Child Health Booklet were utilized. Structured oral interviews captured demographic data, such as their relationship with the child, age, and whether or not other children lived in the household. Interviews were conducted in either English or Kiswahili, whichever the participant felt most comfortable speaking. A research assistant was trained to ask questions and to categorize the responses into the pre-assigned answer choices. A free text option was available if the study team member found that available categories were not appropriate. Retrospective data collection was performed to ascertain health services related data for each child. These data were directly collected from the child’s Mother and Child Health Booklet. A research assistant reviewed the Mother and Child Health Booklets for immunizations, vitamin A supplementation, deworming, and growth monitoring. For immunizations, a research assistant also recorded the child received each dose in each vaccine series and time point or visit for vitamin A supplementation, deworming, and growth monitoring. For all health services data, eligibility for health services was verified by referencing the age of the child, presence at MCH clinic, and timing of service delivery.

Following the conclusion of data collection, health services records extracted from the Mother and Child Health Booklets and responses from the caregiver questionnaires were entered into a Microsoft Excel file. For immunizations, variables were created for each vaccine series to determine whether each child was up-to-date for each vaccine, as defined for each vaccine as the eligibility of child receiving every dose in the series based on age. For example, if a child was 12-weeks old and she received the first three doses of OPV (at birth, 6 weeks, and 10 weeks), she was determined to be up-to-date on OPV despite not receiving the last dose (given at 14-weeks). An additional variable was created to account for every vaccine and series in the immunization schedule. If a child received all vaccines and was up-to-date on all series in the immunization schedule, she was considered up-to-date on all vaccines and fully vaccinated for her age. Similar variables were created for vitamin A supplementation, deworming, and growth monitoring of weight. Height was not accounted for in the growth monitoring variable because only 30% of children had any height measurements recorded.

### Statistical Analysis

Descriptive statistics were used to analyse the responses from the caregiver questionnaire and the proportion of children who received health services and those up-to-date (having received all services they were eligible for). These health services included immunizations, vitamin A supplementation, deworming, and growth monitoring. Additionally, a drop-out rate was calculated for the pentavalent vaccine, which is the proportion of children receiving the first dose in the series but not the third if eligible. Pentavalent drop-out rates are a common way to measure access to services and the capacity of health systems (i.e. MCH clinics) to provide services that require multiple visits (11). Pearson’s chi-square test was applied to determine significant differences in the proportion of males and females up-to-date on all vaccines, vitamin A supplementation, deworming, and weight measurement. Similarly, chi-square was also performed to determine differences in proportion of children fully immunized for age between households with or without other children at home, those who travelled more than 30 minutes to clinic and those who travelled less, different methods of transport, and those who reported barriers in accessing services at the clinic. All analyses were conducted using SPSS (version 24) (12).

## Results

The background characteristics for both the caregivers and children are presented in Table 1. The majority of caregivers were mothers of the children brought to clinic (98.7%), while one caregiver identified as household help. Nearly half of all caregivers (46.2%) were between the ages of 25 – 29 years, and 61.5% had more than one child. Among the children included in the study, gender was evenly distributed (50%) and the mean age was 22.0 weeks (range = 1 – 104). Only four children were over the age of 12 months. MCH clinics in Turbo and Mosoriot had the most caregivers (n=17 and n=16, respectively) followed by Kitale (n=14), Burnt Forest (n=11), Eldoret (n=10), and Webuye (n=10). Over 80% (n=64) of children were first brought to clinic within the first 28 days of life. The most common forms of transportation used to reach clinic were motorbike (38.5%) and walking (37.2%), while the remaining 24.4% of caregivers used matatus, which are privately owned share taxis. The most common reasons for bringing their child to clinic were weight checks (62.8%), immunizations (53.8%), and routine health monitoring (39.7%). Only 20.5% indicated vitamin A supplementation as a reason and 10.3%, listed seeking treatment for illness. When asked what types of services their MCH clinic offered, most caregivers (98.7%) listed growth monitoring, while 48.7% and 37.2% listed immunizations and nutrition, respectively. Only 7.7% listed vitamin A supplementation, and two caregivers (2.3%) said their MCH clinic offered educational health talks and teaching for mothers.

### Immunizations

**Figure 1:**
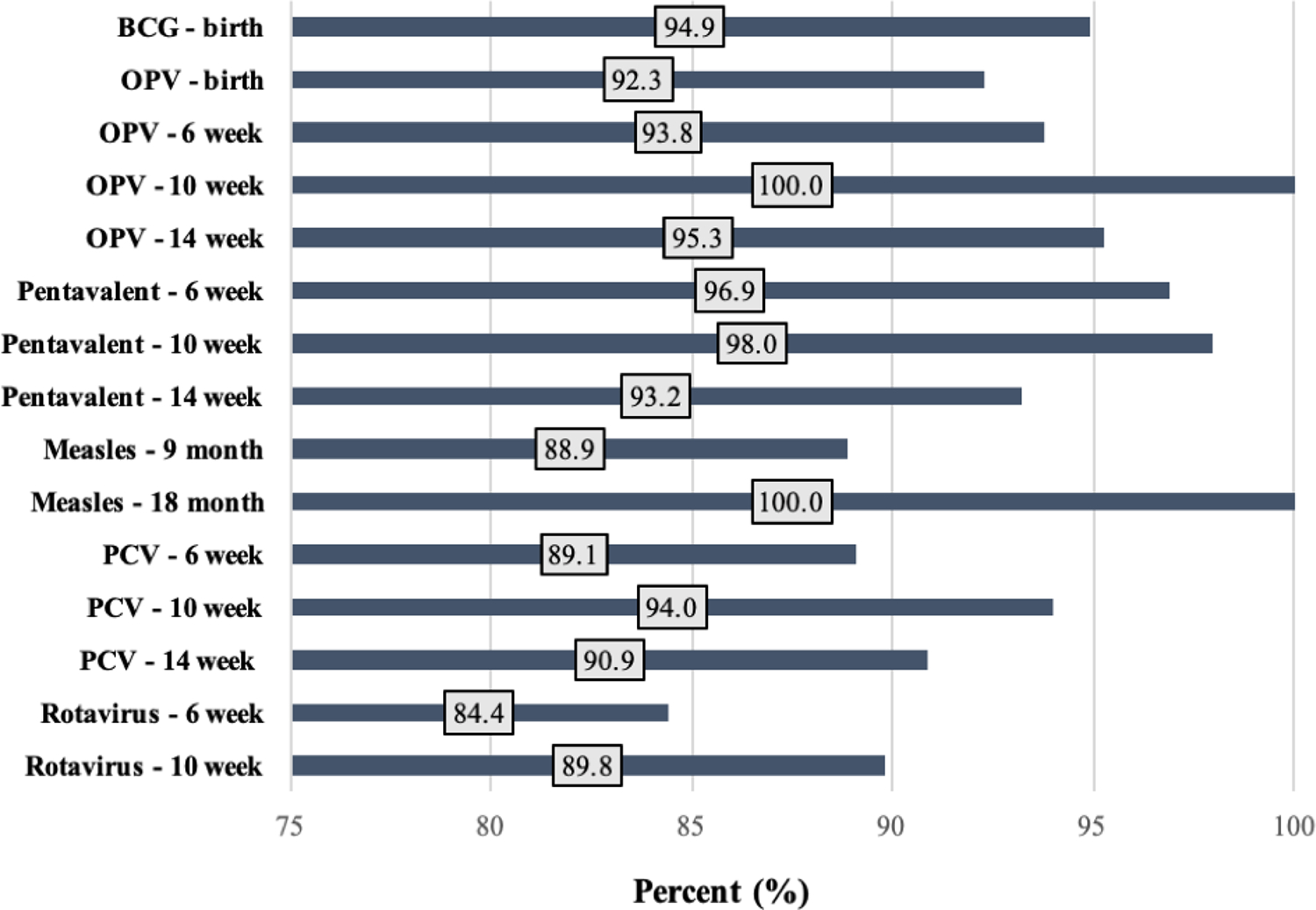
Individual Dose Coverage for Each Vaccine Series

Individual dose coverage for all vaccines ranged from 84.4% to 100%. The third dose of OPV and second dose of measles vaccines had the highest coverage at 100%, while the first dose of rotavirus vaccine had the lowest (84.4%). Out of fifteen total doses of vaccine given, only four doses had coverage below 90% (Fig 1). The drop-out rate for the pentavalent vaccine was 6.7%. Among all vaccines incorporating a 10-week dose in their series (OPV, pentavalent, PCV, and rotavirus), the dose with the highest coverage was the 10-week for all vaccines, regardless of whether it was the second (pentavalent, PCV, rotavirus) or third (OPV) dose in the series.

After accounting for varying child age and eligibility for vaccines, the proportion of children up-to-date on each vaccine was calculated (Table 2). BCG vaccine given at birth had the highest coverage of all vaccines (94.9%), while PCV and rotavirus had the lowest coverage (each 82.8%). The proportions of children up-to-date on pentavalent, OPV, and measles were 92.2%, 85.9%, and 88.9%, respectively. A lesser proportion of females were up-to-date on all vaccines, with the exception of BCG and measles, which had the same coverage as males. Across all MCH sites, Mosoriot was the only site where coverage fell below 80% for any single vaccine. At this site, less than 80% of children were up-to-date on OPV (68.8%), PCV (66.7%), and rotavirus (66.7%).

Slightly less than seventy percent (69.2%) of children were up-to-date on all vaccines and fully vaccinated for their age. The only demographic factor found to be significantly associated with up-to-date vaccination status was gender, as males showed 3.5 times higher odds of being up-to-date for all vaccines compared to females [95% CI 1.256, 9.936]. More than 8 out of every 10 males (82.1%) were up-to-date on all vaccines compared to just 56.4% of females. Differences in the proportion of children up-to-date on all vaccines were also found between MCH sites (Table 2). Among all MCH sites, Kitale had the highest proportion of children up-to-date on all vaccines and fully vaccinated for their age (92.9%). In Eldoret and Webuye, 80% of children were up-to-date on all vaccines, while less than two-thirds were in Turbo (64.7%) and Burnt Forest (63.6%). Mosoriot had the lowest proportion (43.8%).

### Vitamin A and Deworming

Only 35.9% (n=28) of children were eligible for vitamin A supplementation (VAS). Of these, over 75% (n = 21) received VAS at 6 months of age, while 50% received it at 12 months of age. All children received VAS at 18 and 24 months of age (n = 3, n = 1, respectively). Twenty-two (78.6%) of children received every dose of VAS for which they were eligible. VAS coverage was similar between males (80%) and females (77%). Turbo and Burnt Forest were the only MCH sites where a child did not receive VAS. Only 3/7 of children received VAS in Turbo, compared to 3/5 in Burnt Forest. Of the six children eligible to receive deworming medicine at 12 months of age, one two (33.3%) received this medicine. At 12 months, none of the four females eligible received deworming, while both males received this treatment. At 18 months of age, none of the three children eligible received deworming. One child was eligible for deworming at 24 months, and she received the medicine.

### Growth Monitoring

Only one-third (n=26) of children had a birthweight recorded, while 83.1% of children had a weight measurement recorded at six weeks of age. The majority (61.5%) of children had a weight measurement recorded at all MCH visits. Two-thirds of males (66.7%) had a weight recorded at every visit compared to 56.3% of females. Across MCH sites, Eldoret and Kitale had the highest proportions of weight recording, 100% and 90%, respectively, while Burnt Forest (22.2%) and Turbo (30.8%) had the lowest. Only 30.8% (n = 24) of children had a height measurement recorded at one or more MCH clinic visits. None had a height measurement recorded at every MCH visit.

## Discussion

This study aimed to assess the coverage of health services and interventions provided to infants and young children at six MCH clinics located in western Kenya. In this small cross-sectional study, we found that immunization coverage rates were consistent with regional data in Kenya’s national surveys. However, these rates are still below the necessary coverage rates for adequate herd immunity. In addition, we found a gender gap and an inequitable distribution of vaccine coverage across MCH clinics in that males had higher coverage for immunizations and nearly all other services included in the study compared to females.

Nearly 70% of children were up-to-date on all vaccines, which is comparable to national estimates in Kenya reported by the most recent KDHS (5). The KDHS reports 74.9% of Kenyan children are fully vaccinated. Individual vaccine coverage found in our study is also comparable to national estimates as the difference in coverage does not exceed 5% for any single vaccination (5). Subnational vaccine coverage estimates are also consistent with the findings of this study. in In the Rift Valley region of Kenya, the proportion of fully vaccinated children (68.7%) is nearly identical to the findings of the present study (69.2%) (5). In addition, individual vaccine coverage observed in this study more closely resembles the Rift Valley regions than national estimates. Measles coverage at the national (87.1%) and subnational (Rift Valley) (83.1%) levels, as well as in this study (88.9%), are all well below the 95% threshold for achieving herd immunity (13).

The drop-out between the first and third pentavalent vaccine coverage for all MCH clinics was only 6.7%. This drop-out rate is consistent with other studies in Kenya (14), and suggests that the six MCH clinics studied deliver effective immunization services as a whole. However, national and subnational data sources typically use population-based sampling rather than clinic-based, which was used in this study. Recruiting caregivers and their children from the clinic, a population already accessing services, could explain the higher vaccination coverage found in this study. The vaccination coverage rates for the six MCH clinics combined distort the poor coverage in individual clinics, as vaccination coverage varied significantly from site to site. In Mosoriot, less than half of children were considered fully vaccinated for their age. Further investigation is needed to determine factors related to poor vaccination coverage in certain MCH clinics.

Other studies in similar settings found that wealth (15–17), caregiver knowledge of immunization services (16), fewer children in the household (18), skilled birth attendance (17, 19), high health worker performance (16), literacy (16), and parental education (18) was associated with higher vaccination coverage. In this study, factors such as a child’s gender, age of caregiver, number of siblings, type and duration of transportation, and clinic site were assessed; however, only gender was found to be associated with vaccination rates. The small sample size and low power may have influenced the identification of associations within this study.

The finding that males in our study were more likely to be fully vaccinated than females is not consistent with recent KDHS reports and some studies, which showed no difference in coverage rates between males and females (5, 15, 20). These studies were conducted at the national level (5), in an urban setting (20), and in rural western Kenya (15). In our study, we found that males showed over 3.5 times the odds of being fully vaccinated for their age compared to females. A recent study conducted in Nairobi, Kenya also supported the presence of an immunization gender gap, finding that only 65.2% of females were fully vaccinated compared to 73.2% of their male counterparts (21). Similar to the gender disparity found in vaccination coverage, a lesser proportion of females received vitamin A supplementation and growth monitoring. While these differences were not as stark as the gender gap in vaccination coverage, they still raise concern and could indicate that female children do not have the same access to preventive health services as male children. Additional research is needed to ascertain whether caregivers are seeking the same care regardless of their child’s gender in order to develop strategies to improve vaccination coverage in this region of Kenya that target female children to achieve equitable coverage. In future studies, the high coverage rate for vaccines and growth monitoring at 10-weeks of age will be considered as a highly compliant visit in MCH clinics. The completion rate of the 10-week dose was the highest among all vaccines in the series. In addition, the largest proportion of children receiving growth monitoring was at 10-weeks of age as well.

Vitamin A supplementation coverage among the children in the study was higher than findings from the most recent KDHS (5). According to this KDHS, 67.9% of children ages 6–8 months received vitamin A supplementation compared to 78.6% of children receiving vitamin A supplementation within this study at their 6-month visit. Coverage was similar among males and females within the KDHS as well (5). Deworming coverage was lower in the sample than national estimates. Only one-third of children in the present study received deworming medicines in the last twelve months compared to 51% estimated in the most recent KDHS (5). Due to the youth of this study’s sample, it was difficult to accurately measure and compare vitamin A and especially deworming coverage rates to the national levels. The mean age of this sample was just over 5 months and children are not eligible to receive vitamin A supplementation and deworming medicines until 6 and 12 months of age, respectively. Additional data from an older sample of children are needed to provide an estimation of service vitamin A supplementation and deworming coverage.

A limitation of this study is that it was subject to sampling bias as participants were recruited at the MCH clinic. Only caregivers and children who were already present in the MCH clinic were eligible for inclusion in the study. Recruiting participants at the clinic excludes caregivers and children who do not have access to services, encounter barriers in accessing preventive health services, or are disengaged from care for other reasons. This sampling could potentially lead to an overestimation of service coverage because we were unable to account for the children not receiving any services. Additionally, recruiting at each MCH clinic site for just one day could potentially lead to a non-representative sample.

Another limitation was that the sampling strategy might be potential source of overestimating service coverage. For instance, it was difficult to determine whether the caregiver was just arriving or just leaving the clinic at the time of recruitment. Further, when reviewing the Mother and Child Health Booklet, it was assumed that all services were provided on the visit. Therefore, systematic bias was possible within the data source.

Other limitations that could lead to inaccurate coverage estimates include the age of the children in the sample and the sampling strategy. Due to the majority of the children in the sample being under one year of age, it was not possible to determine vaccination coverage in terms of the number of children receiving all required doses. Few children were old enough to have even been eligible to receive all vaccines, so coverage was defined by receipt of eligible vaccines. This could overestimate vaccine coverage because we were unable to account for potential future missed doses. Finally, the small sample size used in this study restricts the representativeness and generalizability of the results.

## Conclusion

Monitoring local coverage of preventive health services for children is a critical component for achieving the Sustainable Development Goals and improving health outcomes and survival among children in Kenya and similar settings in sub-Saharan Africa. We found a significant gender disparity in vaccine coverage, as females in our study had lower odds of being fully immunized compared to males. Further, females were also found to have lower coverage of vitamin A supplementation and growth monitoring. The gender gap found in this study needs further investigation to elucidate what factors are associated with females having significantly lower vaccination coverage than males. This cross-sectional study was small in nature and consequently, limited in its generalizability. However, it is a critical step in understanding how well national and regional data reflect clinic-level data on health service coverage and the factors that impact coverage. This will ultimately get us closer to realizing the Sustainable Development Goals and improving the health of young Kenyan children.

## Figures and Tables

**Table 1.** Participant Characteristics

Variable	n (%)
**Caregiver’s Relationship to Child**	
Mother	77 (98.7)
Household Help	1 (1.3)
**Caregiver’s Age (in years)**	
< 20	1 (1.3)
20 – 24	16 (20.5)
25 – 29	36 (46.2)
30 – 34	17 (21.8)
35 – 39	6 (7.7)
Over 40	2 (2.6)
**Other Children in the Home**	
Yes	48 (61.5)
No	30 (38.5)
**Gender of Child**	
Male	39 (50.0)
Female	39 (50.0)
**Age of Child (in months)**	
< 1	13 (16.7)
1 – 3	29 (37.2)
4 – 6	14 (17.9)
7 – 9	8 (10.3)
10 – 12	10 (12.8)
> 12	4 (5.1)
**Age of Child (in weeks)**	
	Mean: 22.0
**MCH Site Recruitment**	
Eldoret	10
Turbo	17
Webuye	10
Mosoriot	16
Burnt Forest	11
Kitale	14
**Travel Time to MCH Clinic**	
< 30 minutes	27 (34.6)
30 – 59 minutes	28 (35.9)
> 60 minutes	23 (29.5

**Table 2.** Number and Percent of Children Up-to-Date on Vaccines by Gender and MCH Site

	BCG n (%)	OPV n (%)	Pentavalent n (%)	PCV n (%)	Measles n (%)	Rotavirus n (%)	Fully Vaccinated n (%)
**Sex**							
Male	37/39 (94.9)	36/39 (92.3)	33/33 (100)	31/33 (93.9)	8/9 (88.9)	28/33 (84.8)	**32/39 (82.1)**
Female	37/39 (94.9)	31/39 (79.5)	26/31 (83.9)	22/31 (71)	8/9 (88.9)	25/31 (80.6)	**22/39 (56.4)**
**MCH Site**							
Eldoret	10/10 (100)	9/10 (90)	10/10 (100)	9/10 (90)	1/1 (100)	9/10 (90)	**8/10 (80)**
Turbo	14/17 (82.4)	15/17 (88.2)	12/13 (92.3)	11/13 (84.6)	8/8 (100)	11/13 (84.6)	**11/17 (64.7)**
Webuye	10/10 (100)	10/10 (100)	5/5 (100)	5/5 (100)	0/0 (0)	5/5 (100)	**8/10 (80)**
Mosoriot	15/16 (93.8)	11/16 (68.8)	14/15 (93.3)	10/15 (66.7)	2/2 (100)	10/15 (66.7)	**7/16 (43.8)**
Burnt Forest	11/11 (100)	9/11 (81.8)	9/11 (81.8)	9/11 (81.8)	5/5 (100)	10/11 (90.9)	**7/11 (63.6)**
Kitale	14/14 (100)	13/14 (92.9)	9/10 (90)	9/10 (90)	0/0 (0)	8/10 (80)	**13/14 (92.9)**
**Total**	**74/78 (94.9)**	**67/78 (85.9)**	**59/64 (92.2)**	**53/64 (82.8)**	**16/18 (88.9)**	**53/64 (82.8)**	**54/78 (69.2)**

## Data Availability

The datasets used and/or analysed during the current study are available from the corresponding author on reasonable request.

## Abbreviations

AMPATH: Academic Model Providing Access to Healthcare
BCG: Bacillus Calmette-Guerin
Hib: *Haemophilus influenza* type b
KDHS: Kenya Demographic Health Survey
MCH: maternal and child health
OPV: oral polio vaccine
PCV: pneumococcal conjugate vaccine
SSA: sub-Saharan Africa
VAS: vitamin A supplementation
